# Gut dysbiosis is associated with aortic aneurysm formation and progression in Takayasu arteritis

**DOI:** 10.1186/s13075-023-03031-9

**Published:** 2023-03-24

**Authors:** Yusuke Manabe, Tomohiko Ishibashi, Ryotaro Asano, Shuichi Tonomura, Yuichi Maeda, Daisuke Motooka, Jin Ueda, Masahiro Yanagawa, Yuko Edamoto-Taira, Tomomi Chikaishi-Kirino, Takeshi Masaki, Tadakatsu Inagaki, Shota Nakamura, Yoshinori Katada, Makoto Okazawa, Masashi Narazaki, Takeshi Ogo, Atsushi Kumanogoh, Yoshikazu Nakaoka

**Affiliations:** 1 Department of Vascular Physiology, National Cerebral and Cardiovascular Center Research Institute, 6-1, Kishibe-Shimmachi, Suita, Osaka 564-8565 Japan; 2 Department of Respiratory Medicine and Clinical Immunology, Osaka University Graduate School of Medicine, Suita, Japan; 3 Division of Pulmonary Circulation, Department of Cardiovascular Medicine, National Cerebral and Cardiovascular Center, Suita, Japan; 4 Integrated Frontier Research for Medical Science Division, Institute for Open and Transdisciplinary Research Initiatives (OTRI), Osaka University, Suita, Japan; 5 Department of Infection Metagenomics, Research Institute for Microbial Diseases, Osaka University, Suita, Japan; 6 Department of Diagnostic and Interventional Radiology, Osaka University Graduate School of Medicine, Suita, Japan; 7 Department of Respiratory Medicine and Rheumatology, Suita Municipal Hospital, Suita, Japan; 8 Department of Advanced Clinical and Translational Immunology, Osaka University Graduate School of Medicine, Suita, Japan; 9 Department of Immunopathology, WPI, Immunology Frontier Research Center (iFReC), Osaka University, Suita, Japan; 10 Center for Infectious Disease for Education and Research (CiDER), Osaka University, Suita, Japan; 11 Japan Agency for Medical Research and Development – Core Research for Evolutional Science and Technology (AMED–CREST), Osaka University, Suita, Japan; 12 Center for Advanced Modalities and DDS (CAMaD), Osaka University, Suita, Japan; 13 Department of Cardiovascular Medicine, Graduate School of Medicine, Osaka University, Suita, Japan

**Keywords:** Takayasu arteritis, Microbiota, Oral bacteria, Proton pump inhibitor, Aortic aneurysm, Campylobacter

## Abstract

**Background:**

Takayasu arteritis (TAK) is an autoimmune large vessel vasculitis that affects the aorta and its major branches, eventually leading to the development of aortic aneurysm and vascular stenosis or occlusion. This retrospective and prospective study aimed to investigate whether the gut dysbiosis exists in patients with TAK and to identify specific gut microorganisms related to aortic aneurysm formation/progression in TAK.

**Methods:**

We analysed the faecal microbiome of 76 patients with TAK and 56 healthy controls (HCs) using 16S ribosomal RNA sequencing. We examined the relationship between the composition of the gut microbiota and clinical parameters.

**Results:**

The patients with TAK showed an altered gut microbiota with a higher abundance of oral-derived bacteria, such as *Streptococcus* and *Campylobacter*, regardless of the disease activity, than HCs. This increase was significantly associated with the administration of a proton pump inhibitor used for preventing gastric ulcers in patients treated with aspirin and glucocorticoids. Among patients taking a proton pump inhibitor, *Campylobacter* was more frequently detected in those who underwent vascular surgeries and endovascular therapy for aortic dilatation than in those who did not. Among the genus of *Campylobacter*, *Campylobacter gracilis* in the gut microbiome was significantly associated with clinical events related to aortic aneurysm formation/worsening in patients with TAK. In a prospective analysis, patients with a gut microbiome positive for *Campylobacter* were significantly more likely to require interventions for aortic dilatation than those who were negative for *Campylobacter*. Furthermore, patients with TAK who were positive for *C. gracilis* by polymerase chain reaction showed a tendency to have severe aortic aneurysms.

**Conclusions:**

A specific increase in oral-derived *Campylobacter* in the gut may be a novel predictor of aortic aneurysm formation/progression in patients with TAK.

**Supplementary Information:**

The online version contains supplementary material available at 10.1186/s13075-023-03031-9.

## Background

Takayasu arteritis (TAK) is a chronic inflammatory large-vessel vasculitis (LVV), predominantly affecting the aorta and its major branches [1]. TAK most often affects women at approximately 20 years of age [2]. The infiltration of inflammatory cells into large vessels and the release of proinflammatory cytokines, such as interleukin-6 (IL-6), IL-8, IL-17A, IL-18, interferon-γ, and tumour necrosis factor, and acute-phase reactants such as C-reactive protein (CRP) and serum amyloid A, occur during the pathogenesis of TAK [3–6]. Vessel inflammation can result in wall thickening and remodelling, leading to fibrosis, stenosis/occlusion, dilatation/aneurysm, and thrombus formation [7, 8].

Glucocorticoids (GCs) are the mainstay for treating TAK and allow 71% of sustained remission (defined by a use of < 10 mg/day of prednisone) [9]. Despite the high sustained remission rate, more than half of patients with TAK relapse upon tapering or discontinuation of GCs [1, 9]. The recent European Alliance of Associations for Rheumatology (EULAR) and American College of Rheumatology (ACR) guidelines recommend to add non-GC immunosuppressive agents such as disease modifying anti-rheumatic drugs (DMARDs) to spare steroid at the initial treatment for a newly diagnosed patient with TAK [10, 11]. However, there are unmet medical needs for patients with TAK to effectively reduce the dose of GCs in refractory patients with TAK [8, 12].

Tocilizumab is an anti-IL-6 receptor (IL-6R) monoclonal antibody, which blocks IL-6-signaling and reduces inflammation [13]. We and others reported that tocilizumab showed a steroid-sparing effect and improvement in health-related quality of life in patients with refractory TAK [14–17], and tocilizumab was approved for the treatment of TAK in Japan. IL-6 blockade by tocilizumab treatment leads to the suppression of serum inflammation markers such as CRP. Therefore, the disease activity of TAK should be monitored by imaging tests or symptoms of patients in clinical practice. We recently reported the importance of vascular imaging tests, such as computed tomography, in refractory patients with TAK during treatment with tocilizumab through a post-hoc analysis of the TAKT study [18]. In the EULAR recommendation, routine imaging for activity assessment is not recommended for patients in remission, and it is recommended that methods and frequency of imaging study should be decided on an individual basis [10]. Several cases on the deterioration of aortic aneurysms in patients with TAK during tocilizumab therapy have been reported [19, 20]. Therefore, a novel biomarker predicting vascular aggravation, such as dilatation/aneurysm and stenosis/occlusion, in patients with TAK needs to be determined [21].

The close relationship between gut dysbiosis and an altered immune response has been established in recent studies, and such alterations may be associated with the pathogenesis of some autoimmune disorders [22, 23]. The relationship between microorganisms and TAK has been reported previously. Several studies have suggested that different communities of microorganism are found in noninflammatory and inflammatory large-vessel diseases [24]. Many types of microorganisms including bacteria from the oral cavity are detected in tissues of inflammatory aortic aneurysms [25, 26]. The microbiome profile in the blood of patients with LVV has also been reported [27]. In addition, a metagenomic analysis of gut microbiota of patients with TAK was reported recently [28]. However, the relationship between gut microbiome and vascular complications in TAK remains unclear.

In this study, we aimed to 1) investigate whether the gut dysbiosis exists in patients with TAK and 2) identify specific gut microorganisms related to aortic aneurysm formation/progression.

## Methods

Additional details are provided in the Supplementary Material.

### Study design and subjects

In this ambispective (retrospective and prospective) observational study, we enrolled patients with TAK who visited the National Cerebral and Cardiovascular Center, Osaka University Hospital, and Suita Municipal Hospital between February 2020 and December 2021. Faecal samples were collected once at the study enrollment. In the retrospective part of the study, history of aortic aneurysm-related events, namely cardiovascular surgeries or endovascular treatments for aortic aneurysmal dilatation and progression of aortic aneurysms (see Supplementary Methods), was collected. In the prospective part of the study, the time of the faecal sampling was used as a starting point and the occurrence of aortic aneurysm-related events was followed. The diagnosis of TAK was made in accordance with the classification criteria of the ACR in 1990 or the diagnostic Criteria of the Japanese Circulation Society [12, 29]. Approval was obtained from the research ethics committees of the National Cerebral and Cardiovascular Center (R19060-4, M30-072–4), Osaka University Hospital (19,317), and Suita Municipal Hospital (2020-ken 30). Written informed consent was obtained from all of the participants.

### Faecal sample collection and DNA extraction

Faecal sample collection and DNA extraction were performed as described previously with some modifications [30]. Faecal samples were collected using a collection kit containing guanidine solution (TechnoSuruga Laboratory, Shizuoka, Japan). Bacterial DNA was isolated from the samples using the NucleoSpin DNA Stool kit (Macherey–Nagel, Düren, Germany).

### 16S rRNA sequencing, taxonomic classification, and data processing

DNA libraries were prepared according to the Illumina 16S Metagenomic Sequencing Library Preparation Guide with a primer set (27Fmod: 5′-AGR GTT TGA TCM TGG CTC AG-3′ and 338R: 5′-TGC TGC CTC CCG TAG GAG T-3′) targeting the V1–V2 regions of the 16S rRNA gene. Amplicons were subjected to 251-bp paired-end sequencing on the MiSeq system using the MiSeq 500-cycle v2 kit (Illumina, San Diego, CA, USA). Paired-end sequences were analysed with the Qiime2 (version 2021.2, https://qiime2.org) pipeline [31]. Taxon classification of gut bacteria was performed using the SILVA v138 99% OTUs database [32, 33].

### Statistical and bioinformatics analysis

Differences in demographic and clinical information in patients with TAK or HCs were compared using Welch’s t test (when normally distributed), the Mann–Whitney U test (when non-normally distributed), or Fisher’s exact test as appropriate. Kaplan–Meier curves were estimated for aortic aneurysm-related event-free survival. The log-rank test was used to test for differences in the distribution of aortic aneurysm-related events between the groups. Hazard ratios were calculated by the Mantel–Haenszel test. The aortic aneurysm-related event-free survival was defined as the time from faecal sampling to aortic aneurysm-related events defined above. All tests were two-sided. *P* values < 0.05 were considered statistically significant. All analyses were conducted using Prism (v9.3.1, GraphPad Software, San Diego, CA, USA), JMP (v14.2.0, SAS Institute, Cary, NC, USA), and R (v4.2.1, https://www.r-project.org).

## Results

### Characteristics of the study population

In this study, 76 patients with TAK and 56 healthy controls (HCs) were enrolled (Table 1). The median age at sampling was 51 years (interquartile range: 31–68 years) and 67 patients (88.2%) were women in the TAK group. No significant differences in age, sex, or body mass index were found between the TAK and HC groups. Among 19 (25.0%) patients who were newly diagnosed as TAK, 17 patients had not started immunosuppressive treatment at the time of study enrolment, and the median duration of disease was 12 years (interquartile range: 5–30 years; Table 2). According to the Numano scale [34], 42.1% of patients with TAK had type IIb and 34.2% had type V. Active disease (defined as National Institute of Health [NIH] criteria ≥ 2, see Supplementary Methods) was observed in 14 (18.4%) patients [35]. The number of patients who had previously undergone cardiovascular surgery and endovascular treatment was 15 (19.7%) and 9 (11.8%), respectively. Of these, two patients had undergone both cardiovascular surgery and thoracic endovascular aortic repair for aortic aneurysm (Supplementary Table 1). More than half of the patients received GCs (68.4%) and antiplatelets (57.9%), and a proton pump inhibitor (PPI) was administered in 55 (72.4%) patients to prevent gastric ulcers caused by these medications. An anti-IL-6 receptor antibody, tocilizumab, was administered to 30 (39.5%) patients for treatment of TAK at the time of faecal sampling. Trimethoprim-sulfamethoxazole was given in 21 (27.6%) patients for preventing pneumocystis pneumonia.

**Table 1:** Baseline characteristics of the patients with TAK and HCs

	TAK (*n* = 76)	HCs (*n* = 56)	*P* value
Age, years (IQR)	51 (31–68)	48 (34–63)	0.2333
Female, n, %	67, 88.2	48, 85.7	0.794
Body mass index, kg/m^2^ (IQR)	22.0 (20.1–24.7)	21.2 (19.2–23.5)	0.2436
Rheumatic disease			
Rheumatoid arthritis, n, %	4, 5.3	0	0.1366
Sjögren’s syndrome, n, %	1, 1.3	0	> 0.9999
Smoking (current or past) n, %	18, 23.7	11, 19.6	0.6726
Medication			
Anti-hypertension drug, n, %	36, 47.4	6, 10.7	< 0.0001
Statin, n, %	33, 43.4	5, 8.9	< 0.0001
Metformin, n, %	1, 1.3	2, 3.6	0.574
Proton pump inhibitor, n, %	55, 72.4	0	< 0.0001
Antibiotics, n, %	21, 27.6	0	< 0.0001

*HCs* healthy controls, *IQR* interquartile range, *TAK* Takayasu arteritis

**Table 2:** Baseline characteristics of the patients with TAK

	TAK (*n* = 76)
Duration of disease, years (IQR)^a^	12 (5–30)
Newly diagnosed, n, %	19, 25.0
Treatment naïve, n, %	17, 22.4
Inflammatory bowel disease, n, %	8, 10.5
Numano scale	
I, n, %	4, 5.3
IIa, n, %	13, 17.1
IIb, n, %	32, 42.1
III, n, %	0, 0
IV, n, %	1, 1.3
V, n, %	26, 34.2
HLA-B52 positivity, n, %	46, 60.5
CRP, mg/dL, mean ± SD	0.34 ± 1.1
ESR, mm/hour, mean ± SD^b^	14.2 ± 18.8
Modified NIH score ≥ 2^c^, n, %	14, 18.4
Triglyceride, mg/dL, mean ± SD^d^	111 ± 63
LDL cholesterol, mg/dL, mean ± SD^d^	106 ± 29
HDL cholesterol, mg/dL, mean ± SD^d^	66 ± 17
Smoking (current or past), n, %	18, 23.7
Aortic aneurysm, n, %	7, 9.2
Previous cardiovascular surgery, n, %	15, 19.7
Previous endovascular treatment, n, %	9, 11.8
Medication	
Antiplatelet, n, %	44, 57.9
Glucocorticoid, n, %	52, 68.4
Mean dose of prednisolone (mg/day), mean ± SD	5.2 ± 4.9
Methotrexate, n, %	8, 10.5
Azathioprine, n, %	8, 10.5
Cyclosporine, n, %	1, 1.3
5-aminosalicyclic acid, n, %	6, 7.9
Tocilizumab, n, %	30, 39.5
TNF inhibitor, n, %	3, 3.9

*CRP* C reactive protein, *ESR* erythrocyte sedimentation rate, *HDL* high-density lipoprotein, *HLA* human leukocyte antigen, *IQR* interquartile range, *LDL* low-density lipoprotein, *NIH* National Institute of Health, *TAK *Takayasu arteritis, *TNF* tumour necrosis factor

^a^The duration of disease was unknown in 19 patients

^b^Data were missing in nine patients

^c^Considered as patients with active TAK

^d^Data were missing in five patients. A few data of triglyceride were not under fasting condition

### Characteristics of gut microbiome in patients with TAK

**Fig. 1:**
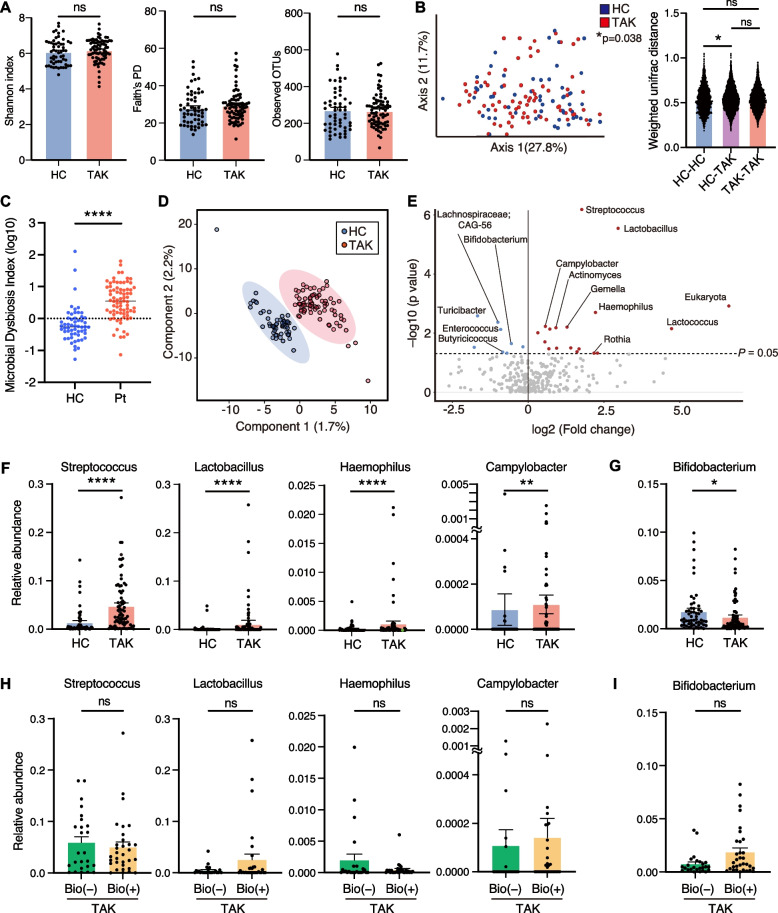
Gut microbial diversity and taxonomy in patients with Takayasu arteritis (TAK) and healthy controls (HCs). **A** Alpha diversity. **B** Principal component analysis (PCoA) at the genus level using the weighted UniFrac distance based on permutational multivariate analysis of variance. **C** Microbial dysbiosis index analysed by the Mann–Whitney U test. **D** Partial least-squares discriminant analysis. **E** Volcano plot of the relative abundance of the gut microbiota at the genus level. The red dots represent increased bacteria and the blue dots represent decreased bacteria in patients with TAK compared with HCs. **F** and **G**, Bar plots of relatively increased bacteria (**F**) and decreased bacteria (**G**) in patients with TAK. In the bar plots, data are shown as the mean ± standard error of the mean. Each dot represents an individual (*n* = 76 for the TAK group and *n* = 56 for the HC group). **H** and **I**, Bar plots of bacteria shown in (**F**) and (**G**), respectively in patients with TAK with or without biological agents (Bio). Each dot represents an individual (*n* = 33 for Bio( +) and *n* = 24 for Bio(–)). ns, not significant. *P < 0.05; **P < 0.01; ****P < 0.0001

We compared the gut microbiome between all patients with TAK and HCs using 16S ribosomal RNA sequencing. Alpha diversity evaluated by the Shannon index, Faith’s phylogenetic diversity, and observed OTUs were similar in the TAK and HC groups (Fig. 1A). Beta diversity evaluated by principal coordinate analysis using the weighted UniFrac distance was different between the groups, which indicated that the gut microbial components differed between the groups (*P* < 0.05; Fig. 1B). We calculated the microbial dysbiosis index and found that the TAK group showed significant gut dysbiosis compared with HC group (*P* < 0.0001; Fig. 1C) [36]. A partial least-squares discriminant analysis (PLS-DA) on the gut microbiome showed different clustering in the observed taxonomy between the two groups (Fig. 1D). Although the relative frequencies at the phylum level were similar between the TAK and HC groups, the TAK group showed a higher abundance of *Streptococcaceae* and a lower abundance of *Bifidobacteriaceae* at the family level (Supplementary Fig. S1A, B). At the genus level, the abundance of *Streptococcus*, *Lactobacillus*, *Haemophilus*, *Campylobacter, Gemella*, and *Actinomyces*, most of which are oral bacteria, was significantly higher in the TAK group than in the HC group. However, the abundance of *Lachnospiraceae* CAG-56 and *Bifidobacterium*, which are lactic acid-producing bacteria, was significantly lower in the TAK group than in the HC group (Fig. 1E–G, Supplementary Fig. S1C, D). These changes were unrelated to the use of biological agents among patients receiving immunosuppressive therapy (Fig. 1H, I). Additionally, because most of the patients with TAK included in this study were inactive, we focused on patients with active TAK (NIH score ≥ 2) and compared their intestinal microbiota with those of HCs. Patients with active TAK showed a similar gut dysbiosis as all patients (Supplementary Figs. S2, S3).

### The effect of drugs on gut microbiome in patients with TAK

**Fig. 2:**
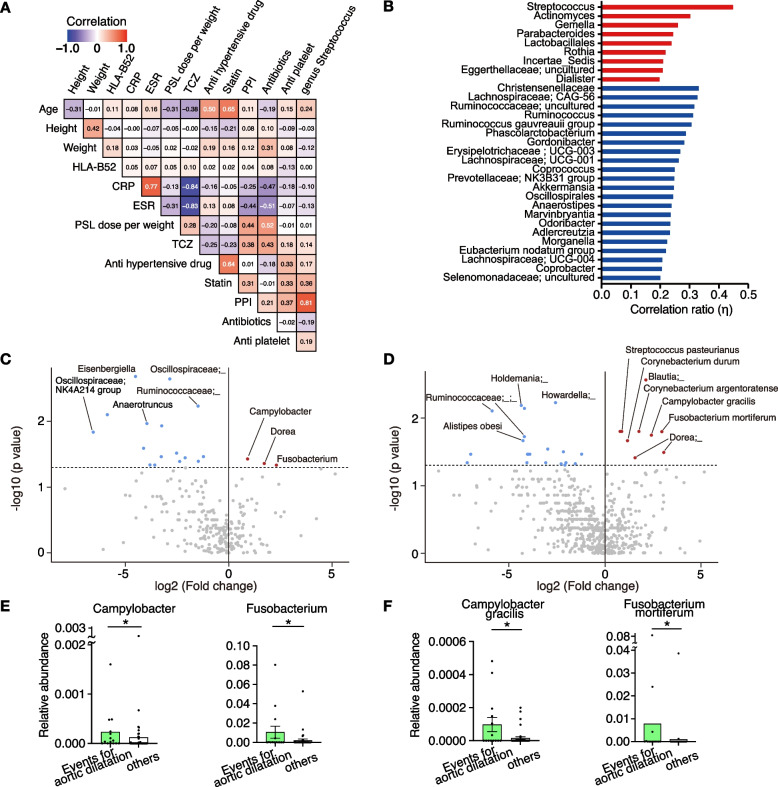
Gut microbiota taxonomy and the risk of vascular events in patients with TAK taking PPIs. **A** Correlation coefficient of clinical parameters and the relative abundance of the genus *Streptococcus*. **B** Correlation coefficient between the gut microbiota at the genus level and PPI use by Spearman’s two-sided rank correlation test (r > 0.2). **C** and **D** Volcano plot of the relative abundance of gut bacteria in patients with TAK taking PPIs with or without events at the genus level (**C**) and species level (**D**). The red dots represent increased bacteria and the blue dots represent decreased bacteria in patients with TAK with events compared with patients with TAK without events. **E** and **F** Relative abundance of *Campylobacter* and *Fusobacterium* at the genus level (**E**), *Campylobacter gracilis* and *Fusobacterium mortiferum* at the species level (**F**) in patients with or without aortic aneurysm-related events. The Mann–Whitney U test was used for analysis. In the bar plots, data are shown as the mean ± standard deviation. Each dot represents an individual. ns, not significant; *P < 0.05

The patients in this study were taking various drugs as in actual clinical practice (Table 2 and Supplementary Table S2). Therefore, we investigated whether these drugs were associated with the relative abundance of the genus *Streptococcus*, which was the most significantly increased genus in the patients compared with HCs (Fig. 1E). We divided patients with TAK into two groups: untreated groups (patients with no history of taking any kinds of immunosuppressive agents) and treated groups. Considering that PPI affects gut microbiota composition [37–39], we also excluded patients taking PPIs from untreated group. First, we compared the gut microbiota among untreated patients (*n* = 11), treated patients (*n* = 59), and HCs (*n* = 56). We calculated the microbial dysbiosis index and found that the index was significantly increased even in untreated patients compared with HCs (Supplementary Fig. S4C). The treated group had higher abundance of oral bacteria such as *Streptococcus*, *Actinomyces*, *Lactobacillus*, *Gemella*, *Haemophilus*, and *Campylobacter* and lower abundance of *Lachnospiraceae* CAG-56 compared with untreated group and HCs in genus level (Supplementary Fig. S4). These results were almost similar to those observed in the comparison between all patients with TAK and HCs (Fig. 1F, G, Supplementary Fig. S1C, D). Since these changes might be affected by the treatment of TAK, we next calculated a correlation coefficient matrix of the relative abundance of the genus *Streptococcus*, laboratory test values, oral medication, and host characteristics (Fig. 2A). The relative abundance of *Streptococcus* in the gut microbiome showed a strong correlation with PPI administration (polyserial correlation coefficient: 0.81). PPIs were taken by 55 patients, and 43 of them received GCs. Other 12 patients were on PPIs without GCs, and 10 of them were treated with antiplatelet or bisphosphonates, both of which are risk factors for gastric ulcer. The remaining two patients were taking PPIs due to gastroesophageal reflux disease rather than prevention of gastric ulcer. This relationship between the relative abundance of genus *Streptococcus* and PPI administration was similarly observed in patients with active TAK (Supplementary Fig. S5A). A comprehensive analysis showed that an increase in oral bacteria, such as *Streptococcus*, *Actinomyces*, and *Lactobacillus*, was strongly correlated with PPI administration (Fig. 2B). PLS-DA showed a clear separation between patients who were taking PPIs (*n* = 55) and HCs (*n* = 56), and the patients without PPI administration (*n* = 21) were plotted between these two groups (Supplementary Fig. S5B). Component 1 of the PLS-DA consisted of oral bacteria, such as *Streptococcus*, *Lactobacillus*, and *Haemophilus* (Supplementary Fig. S5C). The relative abundance of *Streptococcus*, *Gemella*, *Rothia*, and *Campylobacter* was significantly higher in patients with TAK taking PPIs than in those not taking PPIs (Supplementary Fig. S5D). These results suggest that although gut dysbiosis observed in patients with TAK, including an increase in oral bacteria, is affected by PPIs, a similar dysbiosis appears to occur in patients with TAK without PPIs.

### The relationship between gut microbiome and aneurysm in TAK

As the disease status of TAK progresses, patients sometimes require surgery or endovascular intervention for the treatment of vascular complications such as aneurysms. Traditional serum biomarkers such as CRP can be negative in patients treated with biologics such as tocilizumab, and frequent imaging studies are problematic owing to radiation exposure. Therefore, we searched for candidate biomarkers in the gut microbiome to predict these events. We compared the gut microbiome of patients with TAK with and without a prior history of aortic aneurysm-related events, namely cardiovascular surgeries or endovascular treatments for aortic aneurysmal dilatation and progression of aortic aneurysms (see Supplementary Methods). Because the gut microbiota underwent significant changes with PPI administration, this analysis was limited to patients with TAK taking PPIs (Table 3 and Supplementary Table S3). PLS-DA clearly separated the patients with aortic aneurysm-related events (*n* = 14) from those without (*n* = 41) at the genus level (Supplementary Fig. S6A, C) and at the species level (Supplementary Fig. S6B, D). A volcano plot at the genus level showed that the relative abundance of the genera *Campylobacter* and *Fusobacterium* was higher in patients with aortic aneurysm-related events than in those without (Fig. 2C). The abundance of some bacteria, such as *Eisenbergiella* and *Coprococcus*, was lower in patients with aortic aneurysm-related events than in those without (Fig. 2C, Supplementary Fig. S6E). A species-level analysis showed that the increase in the genus *Campylobacter* was mainly due to *Campylobacter gracilis*, which is often found in the oral cavity (Fig. 2D) [40]. Similarly, a species-level analysis showed that the increase in the genus *Fusobacterium* was mainly due to *Fusobacterium mortiferum*, which is also an oral commensal bacteria (Fig. 2D). The genus *Campylobacter* and *C. gracilis* were detected in 22 (40.0%) and 12 (21.8%) patients taking PPIs, respectively. The relative abundance of these bacteria was significantly higher in patients with aortic aneurysm-related events than in those without (Fig. 2E, F). In an analysis of all 76 TAK cases, the relative abundance of *Campylobacter* was associated with PPI administration (Supplementary Fig. S7). The detection rates for the genus *Fusobacterium* (19, 34.5%) and *F. mortiferum* (6, 10.9%) were lower than those for the genus *Campylobacter* and *C. gracilis* (Fig. 2E, F). Notably, the factors which may affect formation/progression of aortic dilatation such as age, body mass index, duration of disease, HLA-B52, inflammation markers, lipid status, and smoking habits were not significantly different between the patients with aortic aneurysm-related events (*n* = 14) and those without (*n* = 41) (Table 3). These results suggest that an increase in the abundance of *Campylobacter* and *Fusobacterium* in the gut microbiota is associated with aortic dilatation, and that these bacteria might be predictive biomarkers of progression of aortic aneurysms.

**Table 3:** Baseline characteristics of patients with TAK taking PPIs with/without surgical and endovascular events

	TAK with events (*n* = 14)	TAK without events (*n* = 41)	*P* value
Age, years (IQR)	60 (29–69)	54 (34–72)	0.8747
Female, n, %	10, 71.4	35, 85.4	0.2552
Body mass index, kg/m^2^ (IQR)	22.7 (21.2–25.2)	22.4 (19.9–24.5)	0.4868
Duration of disease, years (IQR)^a^	12.0 (3.0–48.0)	11.5 (3.8–18.3)	0.6727
Inflammatory bowel disease, n, %	3, 21.4	4, 9.8	0.3537
HLA-B52 positivity, n, %	9, 64.3	24, 58.5	0.7620
CRP, mg/dL, mean ± SD	0.18 ± 0.34	0.24 ± 0.55	0.4880
ESR, mm/hour^b^	9.00 ± 10.08	9.79 ± 10.44	0.7517
Triglyceride, mg/dL, mean ± SD	139 ± 100	104 ± 51	0.3440
LDL cholesterol, mg/dL, mean ± SD	108 ± 44	106 ± 25	0.8945
HDL cholesterol, mg/dL, mean ± SD	67 ± 15	64 ± 18	0.5435
Smoking (current or past) n, %	3, 21.4	10, 24.4	> 0.9999
Medication			
Anti-hypertension drug, n, %	9, 64.3	17, 41.5	0.2154
Statin, n, %	10, 71.4	17, 41.5	0.0683
Metformin, n, %	0, 0	1, 2.4	> 0.9999
Proton pump inhibitor, n, %	14, 100.0	41, 100.0	> 0.9999
Antiplatelet, n, %	6, 42.9	30, 73.2	0.0542
Glucocorticoid, n, %	13, 92.9	30, 73.2	0.1562
Mean dose of prednisolone (mg/day), mean ± SD	7.82 ± 5.32	5.60 ± 4.79	0.1725
Methotrexate, n, %	1, 7.1	7, 17.1	0.6639
Azathioprine, n, %	3, 21.4	4, 9.8	0.3537
Cyclosporine, n, %	1, 7.1	0, 0	0.2545
Tacrolimus, n, %	0, 0	0, 0	> 0.9999
5-aminosalicyclic acid, n, %	2, 14.3	3, 7.3	0.5924
Tocilizumab, n, %	7, 50.0	19, 46.3	> 0.9999
TNF inhibitor, n, %	1, 7.1	2, 4.9	> 0.9999
Antibiotics, n, %	7, 50.0	10, 24.4	0.0983

*CRP* C reactive protein, *ESR* erythrocyte sedimentation rate, *HDL* high-density lipoprotein, *HLA* human leukocyte antigen, *IQR* interquartile range, *LDL* low-density lipoprotein, *PPI* proton pump inhibitor, *TAK* Takayasu arteritis, *TNF* tumour necrosis factor

^a^The duration of disease was unknown in 10 patients

^b^Data were missing in nine patients

### Prediction of aneurysmal complications based on the profiles of gut dysbiosis

**Fig. 3:**
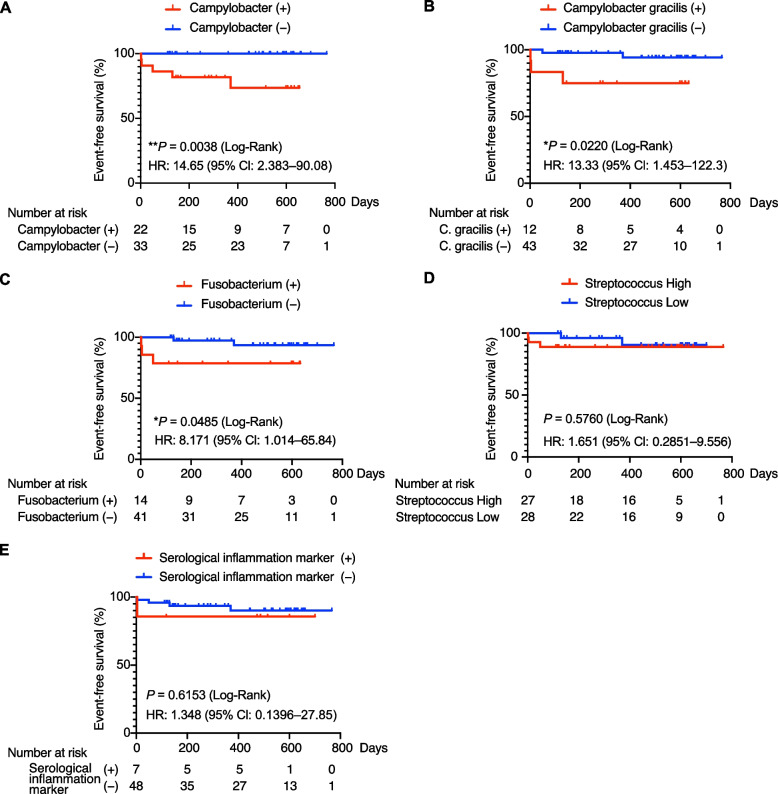
Prospective data of the relationship between gut microbiota taxonomy and patients with aortic aneurysm-related events. **A–C** Aortic aneurysm-related event-free survival in the patients with or without *Campylobacter* (**A**), *Campylobacter gracilis* (**B**), and *Fusobacterium* (**C**). **D** Aortic aneurysm-related event-free survival in the patients with *Streptococcus* abundance higher (High) or lower (Low) than the median. **E** Aortic aneurysm-related event-free survival in the patients who were positive or negative for conventional serological inflammation markers (CRP ≥ 1.0 mg/dL or an erythrocyte sedimentation rate ≥ 30 mm/hour). *P* values were calculated by the log-rank test, and hazard ratios were calculated by the Mantel–Haenszel test

We then investigated whether gut dysbiosis can predict the future progression of aortic aneurysms. Because most of the patients were taking PPIs, we limited this prospective analysis to those taking PPIs. The medium follow-up duration was 16 months (interquartile range, 6.25–19 months). Kaplan–Meier analyses showed that patients who had *Campylobacter* detected in the gut microbiota had a significantly higher incidence of aortic aneurysm-related events, namely surgeries and endovascular treatments due to aortic dilatation, than patients who did not (hazard ratio 14.65, 95% confidence interval 2.383–90.08, *P* < 0.005; Fig. 3A). A similar finding was observed when the patients were stratified by the detection of *C. gracilis* (hazard ratio 13.33, 95% confidence interval 1.453–122.3, *P* < 0.05; Fig. 3B). In addition, patients who had *Fusobacterium* detected in the gut microbiota had a significantly higher incidence of aortic aneurysm-related events than patients who did not (hazard ratio 8.171, 95% confidence interval 1.014–65.84, *P* < 0.05; Fig. 3C). However, there was no difference in aortic aneurysm-related event-free survival according to the relative abundance of *Streptococcus* (Fig. 3D). Conventional serum markers, such as CRP and the erythrocyte sedimentation rate, also failed to predict these events (Fig. 3E).

### Prediction of aneurysmal complications based on PCR analysis of gut microbiome

**Fig. 4:**
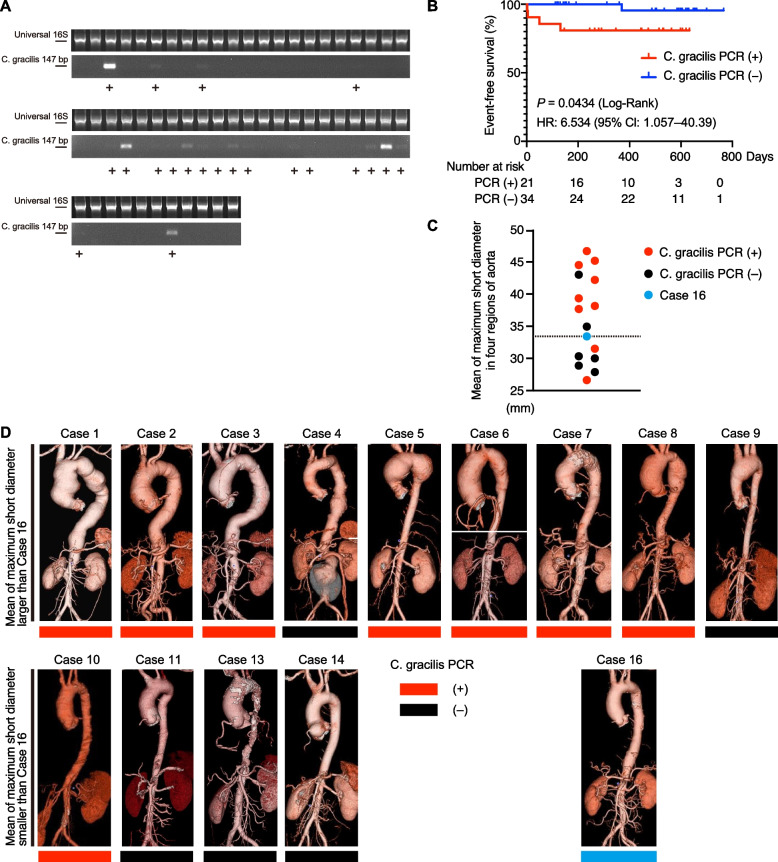
Severity of aortic aneurysms and *C. gracilis* in the gut in patients with TAK. **A** PCR electrophoretic analysis. The upper images show the amplified products of the universal 16S primers (first PCR), and the lower images show those of *C. gracilis* specific 16S primers (second PCR). **B** Aortic aneurysm-related event-free survival in the patients who were positive or negative for *C. gracilis* by PCR. **C** Mean of the maximum short diameters in four regions of the aorta. **D** Three-dimensional computed tomography (3D-CT) images before interventions and positivity of *C. gracilis* by PCR in each patient with TAK who had interventions owing to aortic dilatation. Case 16 is shown as a reference for patients with TAK without interventions. Images in Case 6 are chest CT and abdominal CT. Images of 3D-CT were missing in Cases 12 and 15

We also performed polymerase chain reaction (PCR) to detect *C. gracilis* in the gut microbiota and PCR was positive in 21 patients (Fig. 4A, Supplementary Fig. S8A). Kaplan–Meier analysis showed that patients who were positive for *C. gracilis* by PCR had a significantly higher incidence of aortic aneurysm-related events than those who were negative (hazard ratio 6.534, 95% confidence interval 1.057–40.39, *P* < 0.05; Fig. 4B). We examined whether the severity of aortic aneurysms differed between the patients who were positive for *C. gracilis* by PCR and those who were negative. There was no difference in the proportion of patients treated with tocilizumab with or without *C. gracilis* positivity (Supplementary Fig. S8B). We evaluated computed tomography or magnetic resonance imaging of 15 patients who underwent interventions owing to aortic dilatation before or after stool sample collection. We divided the aorta into four regions and measured the maximum short diameter of each region and calculated the mean diameter (see Supplementary Methods). Based on Case 16, which had the largest mean diameter among patients who did not have an event, nine cases exceeded this value (Fig. 4C). Among these nine patients, seven were positive for *C. gracilis*. The three-dimensional reconstructed aortic images of these patients revealed that patients who were positive for *C. gracilis* by PCR tended to have more severe aortic aneurysms (Fig. 4D, Supplementary Table S4). Smoking, hypertension, male sex, and persistent inflammation are known risk factors for the development of aortic aneurysms in patients with TAK [41]. Case 1 (*C. gracilis*-positive) had a thoracoabdominal aortic aneurysm with the largest mean shortest diameter, despite receiving adequate immunosuppressive therapy and having none of these risk factors. These results suggest that intestinal *C. gracilis* may be a novel tool for predicting aneurysmal formation and progression in TAK.

## Discussion

In this study, we found that the gut microbiota was altered in patients with TAK, and that this alteration may be a predictive marker of aneurysm dilatation. The gut dysbiosis in patients with TAK was characterized by an increased abundance of oral derived bacteria, such as *Streptococcus* and *Campylobacter*. Although this increase may be largely due to treatment including PPIs, comparisons between non-treated patients who received neither immunosuppressive agents nor PPIs and HCs suggested that even non-treated patients exhibited gut dysbiosis. An increased abundance of the genus *Campylobacter* and *C. gracilis* in the gut had a predictive value for surgical and endovascular treatment for dilatational aortic aneurysms in patients who were taking PPIs. In addition, the presence of *C. gracilis* in the gut microbiome was associated with severe aortic aneurysms.

Several studies regarding the relationship between microorganisms and LVV were performed [24, 25, 27]. A study on the blood microbiome of patients with TAK showed a higher abundance of *Bdellovibrio* and *Cytophagaceae*, and a lower abundance of *Staphylococcus* and *Hyphomicrobium* than those in healthy donors [27]. These studies did not mention *Streptococcus* or *Campylobacter*, and whether these increased bacteria in the gut of patients with TAK migrate to aneurysmal lesions via the bloodstream is currently unknown. Recently, a metagenomic analysis of the faecal samples from patients with TAK was reported by Fan et al. [28]. They used shotgun metagenomic sequencing and compared the gut microbiota between patients with TAK and HCs. Interestingly, the finding of increased oral-derived bacteria, such as *Streptococcus*, in the gut of patients with TAK in this previous study is consistent with our results. However, there were some differences between the results of Fan et al.’s study [28] and our study. In our study, the abundance of *Campylobacter* was increased in patients with TAK (Fig. 1E, F). However, Fan et al. reported that the relative abundance of *Campylobacter* was not different between patients with TAK and HCs [28]. One of the reasons for this discrepancy between studies may be that there were only 17 (22.4%) newly diagnosed patients in this study, whereas there was a relatively large number of de novo patients in Fan et al.’s study. Consequently, most of the patients in our study had been already treated with several therapeutic agents and had been taking PPIs for prophylaxis of gastric ulcers (Table 1). Therefore, while Fan et al.’s study mainly described the characteristics of the gut microbiota in patients with naïve TAK, our study included changes related to treatments including PPIs.

Retrospective and prospective analyses of patients with TAK showed a significantly increased abundance of *Campylobacter* in patients with aortic aneurysm-related events. A species-level analysis showed that the increased abundance of *Campylobacter* was due to *C. gracilis* (formerly *Bacteroides gracilis*), which is an anaerobic, gram-negative, and highly antibiotic resistant bacterium primarily found in gingival sulcus [40]. Interestingly, a relationship between *Campylobacter* and cerebral aneurysms was recently reported [42]. According to this previous study, the gut microbiota of patients with subarachnoid haemorrhage (i.e., a ruptured cerebral aneurysm), was different from those with an unruptured cerebral aneurysm, and the abundance of intestinal *Campylobacter*, especially *C. ureolyticus*, was increased in patients with subarachnoid haemorrhage. Although the mechanism by which *Campylobacter* is involved in the aggravation of aneurysms is currently unknown, the use of gnotobiotic mouse model would help us understand the molecular mechanism how alteration of gut microbiome contributes to development of aneurysm in TAK.

We found that the treatment strategy based on traditional inflammatory markers was not useful for predicting the long-term prognosis of patients with TAK. Additionally, even if serological inflammatory markers are negative in patients with TAK treated with tocilizumab, there may be asymptomatic progression of vascular lesions in patients treated with tocilizumab. Currently, most of the biomarkers widely used for TAK are IL-6-dependent inflammation markers (e.g., CRP, the erythrocyte sedimentation rate, and serum amyloid A). In our study, an increase in oral bacteria, such as *Campylobacter*, in the gut was correlated with a poor prognosis of endovascular or cardiovascular surgical treatment. In addition, the increased *Campylobacter* species, *C. gracilis*, was also detectable by PCR. Therefore, these bacteria may be useful as novel biomarkers to predict future aortic aneurysm-related events. Our findings suggest that patients with TAK who are positive for *C. gracilis* in the gut should be followed more intensively by imaging tests than those who are negative.

In this study, an increased abundance of *Streptococcus* and *Campylobacter* in the gut was associated with the administration of PPIs (Fig. 2A, Supplementary Fig. S6). PPIs suppress gastric acid secretion, leading to an increased abundance of oral bacteria in gut [37–39]. In patients with TAK, a PPI is often administered as prophylaxis against complications of therapeutic agents. Low-dose aspirin, which increase the risk of gastric ulcers [43, 44], is recommended in various guidelines, including in Japan, to lower the risk of ischemic events in patients with LVV [12, 45–47]. However, the routine use of low-dose aspirin in patients with LVV has been reconsidered because of the lack of solid evidence [10, 11]. The long-term use of PPIs is associated with various adverse effects, such as anaemia, bone fractures, dementia, diarrhea, trace element deficiencies, and malignant tumours [48–50]. Additionally, PPI-induced alterations in the gut microbiota, such as those observed in this study, may also lead to *Clostridium difficile* colitis [48–50]. An increased mortality rate was also reported in cirrhotic patients taking PPIs who had an increased abundance of *Streptococcus salivarius* in the gut [51]. Our findings indicated that increased oral bacteria in the gut due to PPI administration may have a role in exacerbation of aortic aneurysms. In some cases, temporary discontinuation or switching to other antiplatelet agents other than low-dose aspirin should be considered.

Our study has several limitations. First, the oral microbiota was not analysed in this study. Further studies are required to clarify whether increased oral bacteria in the gut is associated with changes in the oral microbiota. Second, various factors that may affect the composition of the gut microbiota, such as medication, diet, preservation of stools, and DNA extraction methods, should be verified in further studies. Third, our study has some missing data such as ESR (9/76 were missing) and disease duration (19/76 were missing). Finally, this study was based on data from limited medical centres and a single ethnic group. Therefore, a larger study with multiple centres and multiple ethnicities is necessary to validate the results of this study.

## Conclusion

In conclusion, this study suggests that there is gut dysbiosis with an increase in oral bacteria in patients with TAK, and that this alteration is associated with formation and progression of aortic aneurysms.

## Supplementary Information

**Additional file 1.**

## Data Availability

The datasets used in and analysed in this study are available from the corresponding author on reasonable request.

## Abbreviations

CRP: C-reactive protein
GC: Glucocorticoid
HCs: Healthy controls
IL: Interleukin
LVV: Large-vessel vasculitis
NIH: National Institute of Health
OTU: Operational taxonomic unit
PCR: Polymerase chain reaction
PLS-DA: Partial least-squares discriminant analysis
PPI: Proton pump inhibitor
TAK: Takayasu arteritis
