# Supplementary material for: Gut dysbiosis is associated with aortic aneurysm formation and progression in Takayasu arteritis
Source: Arthritis Res Ther. 2023 Mar 24;25:46. doi: 10.1186/s13075-023-03031-9 (PMC10037851; doi:10.1186/s13075-023-03031-9)
Supplement: Supplementary file 1 — Additional file 1. [file 13075_2023_3031_MOESM1_ESM.pdf]

Manabe et al.

Gut dysbiosis is associated with aortic aneurysm formation and progression in Takayasu arteritis

SUPPLEMENTAL MATERIAL

Detailed Methods

Study design and subjects

In this ambispective (retrospective and prospective) observational study, we enrolled patients with TAK who visited the National Cerebral and Cardiovascular Center, Osaka University Hospital, and Suita Municipal Hospital between February 2020 and December 2021. Seventy-six patients were enrolled. The inclusion criteria for patients with TAK were as follows: diagnosis of TAK according to the classification criteria of the American College of Rheumatology in 1990 or the diagnostic Criteria of the Japanese Circulation Society [1, 2]. The control subjects were selected from age- and sex-matched subjects among healthy subjects who were enrolled in a study approved by the Research Ethics Committee of the National Cerebral and Cardiovascular Center (M30-060-7). The inclusion criteria for healthy subjects were defined as follows: not having received any antibiotic within 1 month of stool collection, not having a history of autoimmune diseases or untreated malignant neoplasms, and not having extreme diets, such as strict vegetarians. Finally, 56 healthy subjects were enrolled. This study was conducted in accordance with the principles of the Declaration of Helsinki. Approval was obtained from the research ethics committees of the National Cerebral and Cardiovascular Center (R19060-4, M30-072-4), Osaka University Hospital (19317), and Suita Municipal Hospital (2020-ken 30). Written informed consent was obtained from all of the participants.

Data collection

The following information was collected through the medical records and a personal questionnaire: age, sex, height, body weight, body mass index, duration of disease, distribution of vasculitis lesions, blood test results, medical history, medical treatments, and demographic profiles. Human leukocyte antigen alleles were analysed using WAKflow (Wakunaga Pharmaceutical, Hiroshima, Japan) and the Bio-Plex 200 system (Bio-Rad, Hercules, CA, USA) when they could not be collected from the medical records. Data from blood tests and imaging data, such as magnetic resonance imaging, computed tomography, ultrasonography, and fluorodeoxyglucose-position emission tomography, were selected on the date closest to the date of stool collection. Aortic aneurysm-related events were defined as follows: (1) cardiovascular surgeries or endovascular treatments for aortic aneurysmal dilatation caused by TAK; and (2) progression of aortic aneurysms with a maximum short diameter of ≥ 55 mm, which is an indication for surgery [3, 4]. The maximum short diameter of the aortic aneurysm was measured by using imaging data of computed tomography or magnetic resonance imaging. To calculate the mean value of the aortic diameter, we also separated the aorta into four regions which were defined as follows: the ascending aorta (from the sinotubular junction to the origin of the innominate artery), aortic arch (from the origin of the innominate artery to the isthmus, approximately 2 cm distal to the left subclavian artery), descending thoracic aorta (from the isthmus to the aorta at the diaphragm, 2 cm above the origin of the celiac axis), and abdominal aorta (distal aorta of the descending thoracic aorta) [5]. We then measured the maximum short diameter of the aortic aneurysm in each region. If an aortic aneurysm was not detected, we measured the maximum short diameter in certain points as recommended [5]. An aortic aneurysm was defined as a maximum short diameter of > 45 mm in the thoracic aorta and > 30 mm in the abdominal aorta [6]. The maximum short diameter of the aorta was measured by two independent researchers who did not know the results of the gut microbiota analysis.

Disease activity was defined by a modified score derived from National Institutes of Health criteria [7] according to the presence of the following criteria: a new ischemic vascular sign (i.e., claudication, dyspnea, ischemic pain in the chest, abdomen, or limbs, bruit or asymmetry in the pulse or blood pressure, carotidynia, and pulse abolition); a new arterial lesion or worsening of pre-existing lesions on imaging; systemic clinical features (i.e., fever, weight loss, generalized malaise, myalgia, joint pain, fatigability, and hypertension in younger patients); and an elevation in biological markers (CRP concentrations ≥ 1 mg/dL or an erythrocyte sedimentation rate ≥ 30 mm/hour) [8]. Disease was considered active if the modified National Institutes of Health score was ≥ 2, and inactive otherwise.

Faecal sample collection and DNA extraction

Faecal sample collection and DNA extraction were performed as described previously with some modifications [9]. Faecal samples were collected using a collection kit containing guanidine solution (TechnoSuruga Laboratory, Shizuoka, Japan) and transported to our laboratory. They were stored at 4°C until DNA extraction. Bacterial DNA was isolated from the samples using the NucleoSpin DNA Stool kit (Macherey-Nagel, Düren, Germany) by following the instruction manual. The DNA was stored at −20°C until 16S ribosomal RNA (rRNA) gene sequencing.

16S rRNA sequencing, taxonomic classification, and data processing

DNA libraries were prepared according to the Illumina 16S Metagenomic Sequencing Library Preparation Guide with a primer set (27Fmod: 5′-AGR GTT TGA TCM TGG CTC AG-3′ and 338R: 5′-TGC TGC CTC CCG TAG GAG T-3′) targeting the V1–V2 regions of the 16S rRNA gene. Amplicons were subjected to 251-bp paired-end sequencing on the MiSeq system using the MiSeq 500-cycle v2 kit (Illumina, San Diego, CA, USA). Paired-end sequences were

analysed with the Qiime2 (version 2021.2, https://qiime2.org) pipeline [10]. Sequences were demultiplexed and analysed on the DADA2 pipeline. Taxon classification of gut bacteria was performed using the SILVA v138 99% OTUs database [11, 12].

PCR assay specific for C. *gracilis*

A PCR assay specific for *C. gracilis* was performed as described previously with some modifications [13]. The extracted DNA was used in the first PCR reaction using universal 16S rRNA primers. PCR amplification was performed using KOD FX DNA polymerase (Toyobo, Osaka, Japan) containing 10 µL PCR reaction mixture (2× KOD FX Buffer 5 µL, 2 mM dNTPs 2 µL, 10 µM primer 0.6 µL, 5 ng/µL template DNA 1 µL, 1 U/µL KOD FX DNA polymerase 0.2 µL, and cresol red 1.2 µL). In the nested *C. gracilis* specific reaction, 1 µL of ×1/1000 universal reaction was used as a template. The following primers were used: universal 16S rRNA, 5′-AGA GTT TGA TCC TGG CTC AG-3′ (forward) and 5′-ACG GCT ACC TTG TTA CGA CTT-3′ (reverse); and *C. gracilis*, 5′-AAC GGA ATT TAA GAG AGC TT-3′ (forward) and 5′-CTT TCC CGA TTT ATC TTA TG-3′ (reverse). A thermal cycler was used with the following program: 94°C for 3 min, followed by 32 cycles of 30 sec at 94°C, 30 sec at 60°C, and 40 sec at 72°C, and a final extension at 72°C for 3 min. PCR products were visualized on an agarose gel by using standard protocols.

Bioinformatics analysis

The alpha diversity indices were estimated by the Shannon index, Faith's phylogenetic diversity, and observed OTUs. The beta diversity was estimated by using principal coordinate analysis to evaluate the differences in bacterial composition between patients with TAK and HCs. The microbial dysbiosis index was determined as log10 of the total abundance of increased bacteria in patients with TAK divided by the total abundance of decreased bacteria

in patients with TAK [14, 15]. PLS-DA was performed and the volcano plot was constructed using MetaboAnalyst 5.0 to further analyse the differences in gut microbiome taxonomy between the groups [16].

Supplementary references

1 Isobe M, Amano K, Arimura Y, et al. JCS 2017 Guideline on Management of Vasculitis Syndrome- Digest Version. Circ J 2020;84(2):299-359.

2 Arend WP, Michel BA, Bloch DA, et al. The American College of Rheumatology 1990 criteria for the classification of Takayasu arteritis. Arthritis Rheum 1990;33(8):1129-34.

3 Mortality results for randomised controlled trial of early elective surgery or ultrasonographic surveillance for small abdominal aortic aneurysms. The UK Small Aneurysm Trial Participants. Lancet 1998;352(9141):1649-55.

4 Svensson LG, Kouchoukos NT, Miller DC, et al. Expert consensus document on the treatment of descending thoracic aortic disease using endovascular stent-grafts. Ann Thorac Surg 2008;85(1 Suppl):S1-41.

5 Hiratzka LF, Bakris GL, Beckman JA, et al. 2010 ACCF/AHA/AATS/ACR/ASA/SCA/SCAI/SIR/STS/SVM guidelines for the diagnosis and management of patients with Thoracic Aortic Disease: a report of the American College of Cardiology Foundation/American Heart Association Task Force on Practice Guidelines, American Association for Thoracic Surgery, American College of Radiology, American Stroke Association, Society of Cardiovascular Anesthesiologists, Society for Cardiovascular Angiography and Interventions, Society of Interventional Radiology, Society of Thoracic Surgeons, and Society for Vascular Medicine. Circulation 2010;121(13):e266-369.

6 Erbel R, Aboyans V, Boileau C, et al. 2014 ESC Guidelines on the diagnosis and treatment of aortic diseases: Document covering acute and chronic aortic diseases of the thoracic and abdominal aorta of the adult. The Task Force for the Diagnosis and

Treatment of Aortic Diseases of the European Society of Cardiology (ESC). Eur Heart J 2014;35(41):2873-926.

7 Kerr GS, Hallahan CW, Giordano J, et al. Takayasu arteritis. Ann Intern Med 1994;120(11):919-29.

8 Nakaoka Y, Isobe M, Takei S, et al. Efficacy and safety of tocilizumab in patients with refractory Takayasu arteritis: results from a randomised, double-blind, placebo-controlled, phase 3 trial in Japan (the TAKT study). Ann Rheum Dis 2018;77(3):348-54.

9 Kameoka S, Motooka D, Watanabe S, et al. Benchmark of 16S rRNA gene amplicon sequencing using Japanese gut microbiome data from the V1-V2 and V3-V4 primer sets. BMC Genomics 2021;22(1):527.

10 Bolyen E, Rideout JR, Dillon MR, et al. Reproducible, interactive, scalable and extensible microbiome data science using QIIME 2. Nat Biotechnol 2019;37(8):852-7.

11 Robeson 2nd MS, O'Rourke DR, Kaehler BD, et al. RESCRIPt: Reproducible sequence taxonomy reference database management for the masses. bioRxiv 2020.10.05.326504.

12 Bokulich NA, Kaehler BD, Rideout JR, et al. Optimizing taxonomic classification of marker-gene amplicon sequences with QIIME 2's q2-feature-classifier plugin. Microbiome 2018;6(1):90.

13 Siqueira JF, Jr., Rocas IN. Campylobacter gracilis and Campylobacter rectus in primary endodontic infections. Int Endod J 2003;36(3):174-80.

14 Gevers D, Kugathasan S, Denson LA, et al. The treatment-naive microbiome in new-onset Crohn's disease. Cell Host Microbe 2014;15(3):382-92.

15 Lin YT, Lin TY, Hung SC, et al. Anti-Acid Drug Treatment Induces Changes in the Gut Microbiome Composition of Hemodialysis Patients. Microorganisms 2021;9(2).

16 Chong J, Xia J. MetaboAnalystR: an R package for flexible and reproducible analysis of metabolomics data. Bioinformatics 2018;34(24):4313-4.

Supplementary Table S1. Specific details of prior history of cardiovascular surgeries or endovascular treatments to the legions of damaged vessels in patients with TAK at the time of stool sampling

Variables	Number of TAK patients (n = 76)<br>n (%)
Without events	54 (71.1)
With surgeries or endovascular treatments	22 (28.9)
Cardiovascular surgery	15 (19.7)
Aortic replacement	
Total arch replacement	5 (6.6)
Hemiarch replacement	1 (1.3)
Proximal aortic repair	2 (2.6)
Repairment of TAAA	1 (1.3)
Y graft replacement	1 (1.3)
Valve replacement	
aortic valve replacement	6 (7.9)
Aortic root replacement (Bentall)	2 (2.6)
Coronary artery bypass grafting (CABG)	2 (2.6)
Bypass surgery	2 (2.6)
Reparing of first branches of aorta (SCA, CCA)	1 (1.3)
Renal transplantation	1 (1.3)
Endovascular treatment	9 (11.8)
Thoracic endovascular aortic repair (TEVAR)	2 (2.6)
PTA (renal artery, brachiocephalic trunk)	2 (2.6)
Embolization	
Coil embolization for aneurysm of ICA	1 (1.3)
Bronchial arterial embolization (BAE)	1 (1.3)
Percutaneous coronary intervention (PCI)	2 (2.6)
Stenting (ICA, SCA, CCA)	2 (2.6)

Values are expressed as numbers and percentages.

CCA, common carotid artery; ICA, internal carotid artery; PTA, percutaneous transluminal angioplasty; SCA, subclavian artery; TAAA, thracoabdominal aortic aneurysm; TAK, Takayasu arteritis.

Supplementary Table S2. Baseline characteristics of the patients with active TAK and HCs

	active TAK (n = 14)	HCs (n = 56)	*P* value
Age, years (IQR)	50 (28–69)	48 (34–63)	0.9971
Female, n, %	13, 92.9	48, 85.7	0.6755
Body mass index, kg/m² (IQR)	22.8 (20.1–25.1)	21.2 (19.2–23.5)	0.3165
Duration of disease, years (IQR)*	2.5 (0.5–14.3)	-	N/A
HLA-B52 positivity, n, %	5, 35.7	-	N/A
CRP, mg/dL, mean ± SD	1.3 ± 2.2	-	N/A
ESR, mm/hour, mean ± SD	34.9 ± 29.6	-	N/A
Smoking (current or past) n, %	5, 35.7	11, 19.6	0.2843
Medication			
Anti hypertension drug, n, %	8, 57.1	6, 10.7	0.0005
Statin, n, %	7, 50.0	5, 8.9	0.0013
Metformin, n, %	1, 7.1	2, 3.6	0.4936
Proton pump inhibitor, n, %	8, 57.1	0, 0	< 0.0001
Antiplatelet, n, %	10, 71.4	0, 0	< 0.0001
Glucocorticoid, n, %	5, 35.7	-	N/A
Mean dose of prednisolone (mg/day), mean ± SD	12.1 ± 3.5	-	N/A
Methotrexate, n, %	3, 21.4	-	N/A
Azathioprine, n, %	2, 14.3	-	N/A
Cyclosporine, n, %	0, 0	-	N/A
Tacrolimus, n, %	0, 0	-	N/A
5-aminosalicyclic acid, n, %	0, 0	-	N/A
Tocilizumab, n, %	2, 14.3	-	N/A
TNF inhibitor, n, %	0, 0	-	N/A
Antibiotics, n, %	2, 14.3	0, 0	0.0377

CRP, C reactive protein; ESR, erythrocyte sedimentation rate; HCs, healthy controls; HLA, human leukocyte antigen; IQR, interquartile range; N/A, not assessed; TAK, Takayasu arteritis; TNF, tumour necrosis factor.
*The duration of disease was unknown in 4 patients.

Supplementary Table S3: Specific details of prior history of aortic aneurysm-related events in patients with TAK taking PPIs (n = 55) at the time of stool sampling

Variables	n (%)
Without events	41 (74.5)
With events of dilated legions	14 (25.5)
Aortic aneurysm with a maximum short diameter of ≥ 55 mm	7 (12.7)
Cardiovascular surgery	
Aortic replacement	
Total arch replacement	5 (9.1)
Hemiarch replacement	1 (1.8)
Proximal aortic repair	2 (3.6)
Repairment of TAAA	1 (1.8)
Y graft replacement	1 (1.8)
Valve replacement	
aortic valve replacement	6 (10.9)
Aortic root replacement (Bentall)	2 (3.6)
Reparing of first branches of aorta (SCA, CCA)	1 (1.8)
Endovascular treatment	
Thoracic endovascular aortic repair (TEVAR)	2 (3.6)
Embolization	
Coil embolization for aneurysm of ICA	1 (1.8)

Values are expressed as numbers and percentages.
CCA, common carotid artery; ICA, internal carotid artery; PPI, proton pump inhibitor; PTA, percutaneous transluminal angioplasty; SCA, subclavian artery; TAAA, thracoabdominal aortic aneurysm; TAK, Takayasu arteritis.

Supplementary Table S4. Baseline characteristic features in patietns with TAK taking PPIs who underwent cardiovascular surgeries or EVTs for aortic dilated lesions at the time of the latest surgery.

Case	Age	Sex	Duration of disease	HLA-B52	Smoking	HT	Inflammation	PSL dose (mg/day)	Immuno-suppressants	TCZ	Latest surgical procedure
1	26	f	6 years	+	−	−	−	12	+	+	TAR + cET
2	61	m	unknown	−	+	+	+	−	−	−	Bentall + PAR
3	76	f	unknown	−	−	+	−	7.5	−	−	TAAA repairment for TAR
4	56	f	28 years	−	+	+	−	10	−	−	TEVAR
5	29	f	3 years	+	−	−	−	16	+	+	Extended TAR + right SCA/CCA repair
6	37	m	8 years	−	−	+	−	20	−	−	TEVAR
7	44	m	unknown	+	−	+	+	−	−	−	AVR + TAR
8	28	f	2 years	−	−	+	−	13	−	−	Bentall + TAR
9	23	m	unknown	+	−	−	+	−	−	−	TAR
10	54	f	unknown	+	−	+	+	−	−	−	AVR
11	21	f	unknown	−	−	−	−	10	+	−	CABG + AVR + ascending aortic replacement
12	54	f	32 years	+	−	+	unknown	+ (unknown)	−	−	AVR
13	51	f	33 years	+	+	unknown	−	−	−	−	AVR
14	37	f	11 years	+	−	−	−	6	−	+	AVR
15	60	f	1 year	+	−	+	−	10	+	−	Coil embolization for aneurysm of left ICA
16*	43	m	9 years	+	+	+	−	5	−	+	−

AVR, aortic valve replacement; CABG, coronary artery bypass grafting; CCA, common carotid artery; cET, classical elephant trunk; HLA, human leukocyte antigen; HT, hypertension; ICA, internal carotid artery; MVP, mitral valve plasty; PAR, Part of aortic arch replacement; PPI, proton pump inhibitor; PSL, prednisolone; SCA, subclavian artery; TAAA, thoracoabdominal aortic aneurysm; TAK, Takayasu arteritis; TAR, total aortic replacement; TCZ, tocilizumab; TEVAR, thoracic endovascular aortic repair.

*Parameters of case 16 were collected at the time when 3D-CT imaging was performed.

Supplementary Figure S1

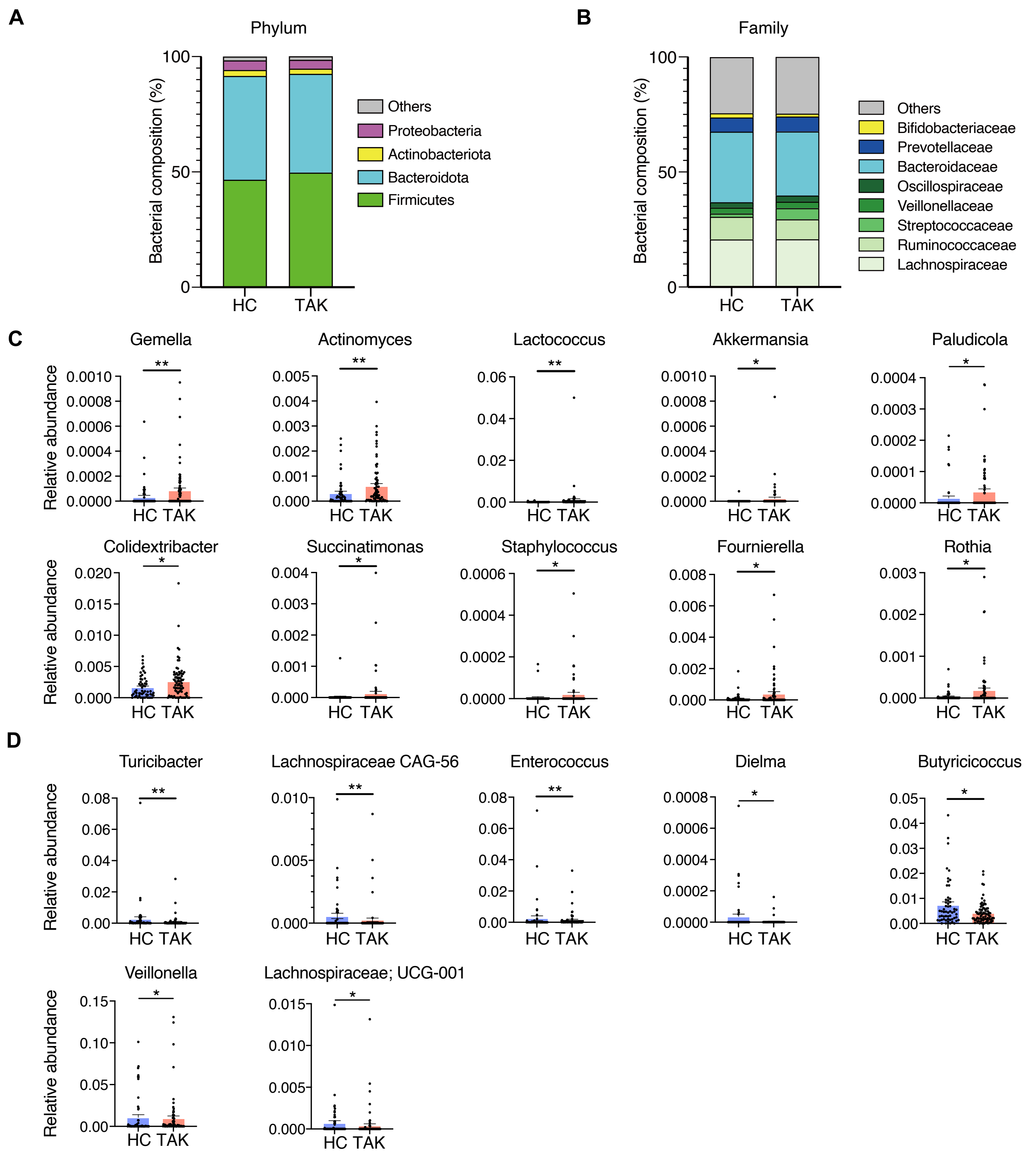

Supplementary Figure S2

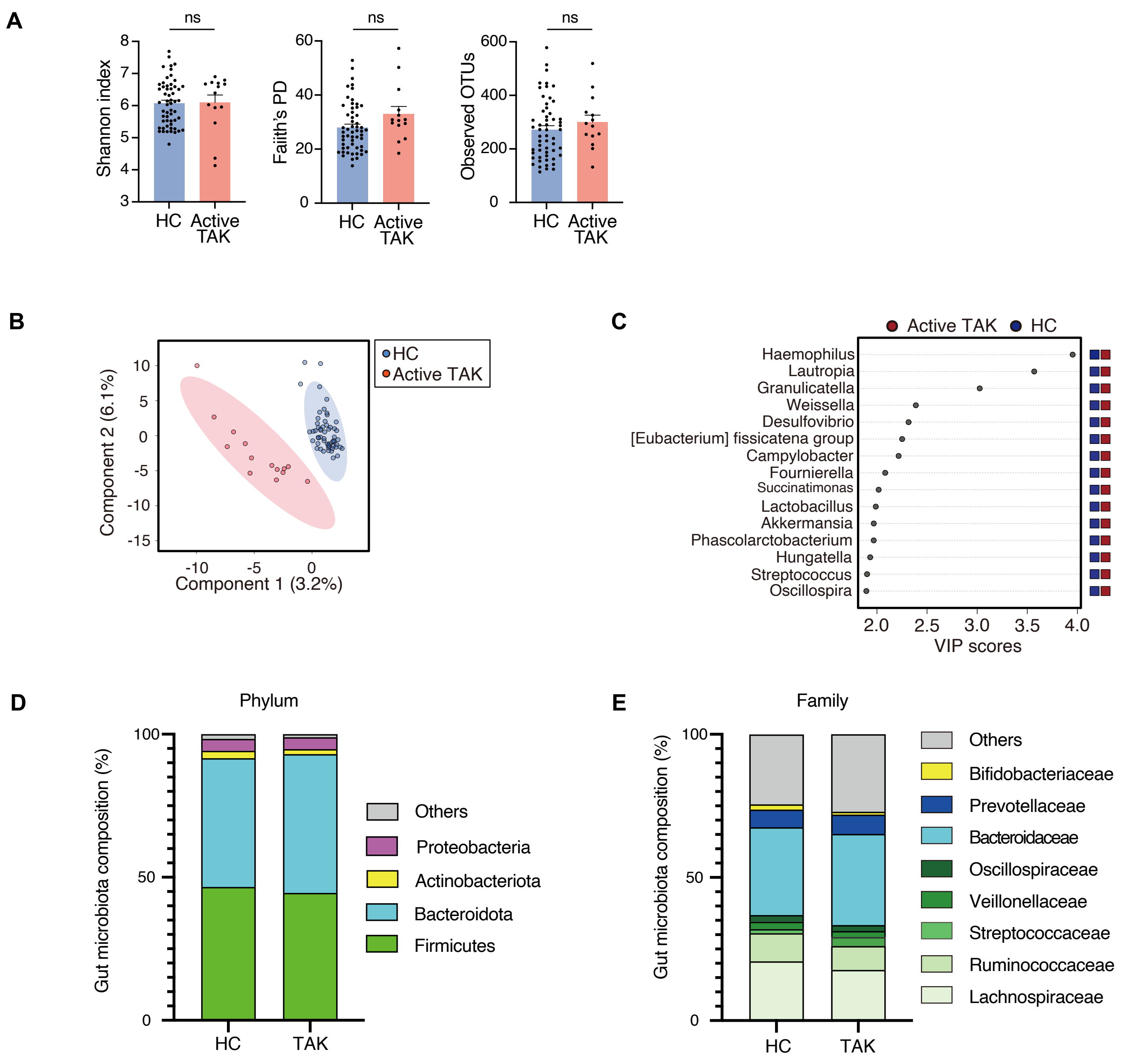

Supplementary Figure S3

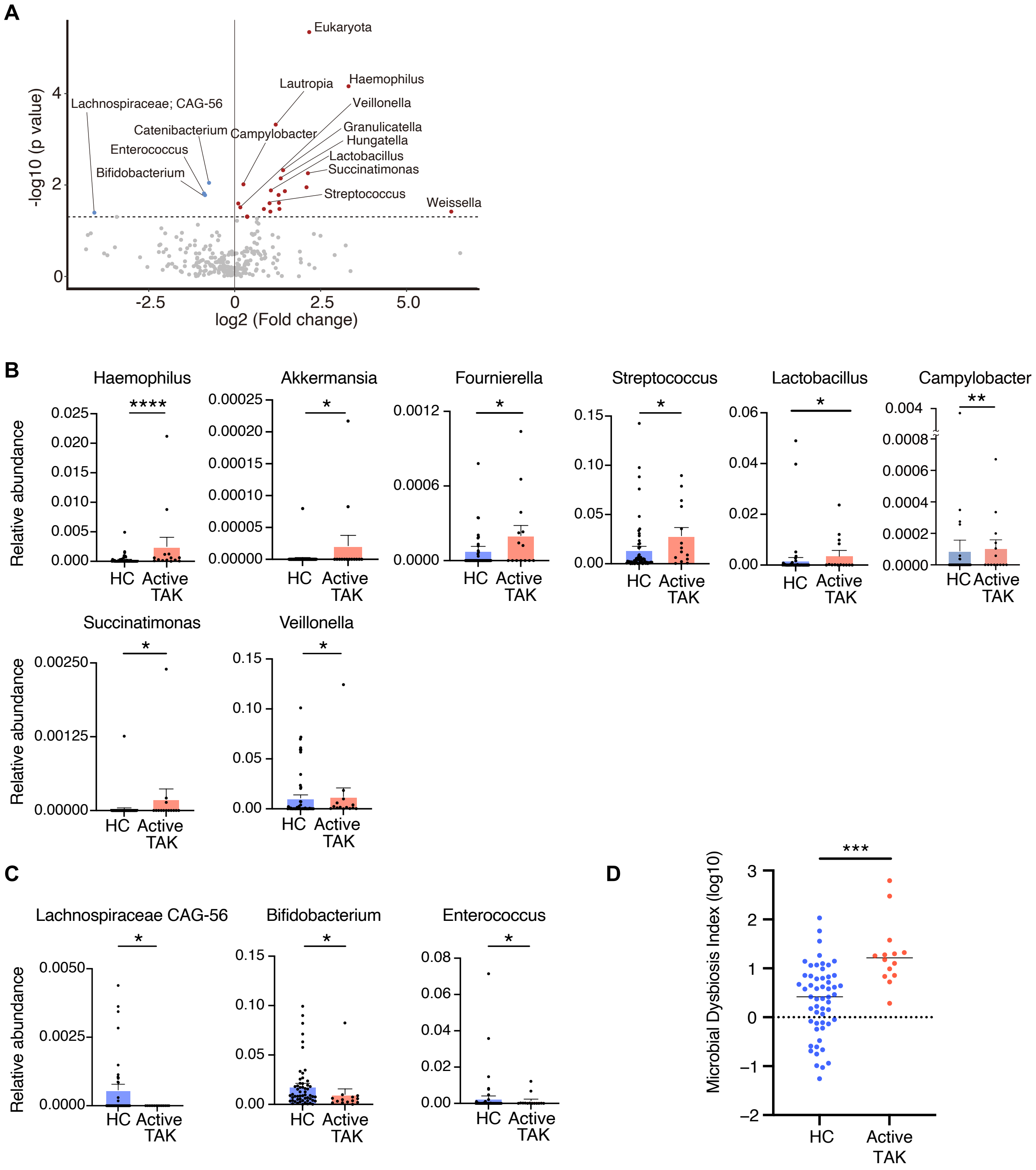

Supplementary Figure S4

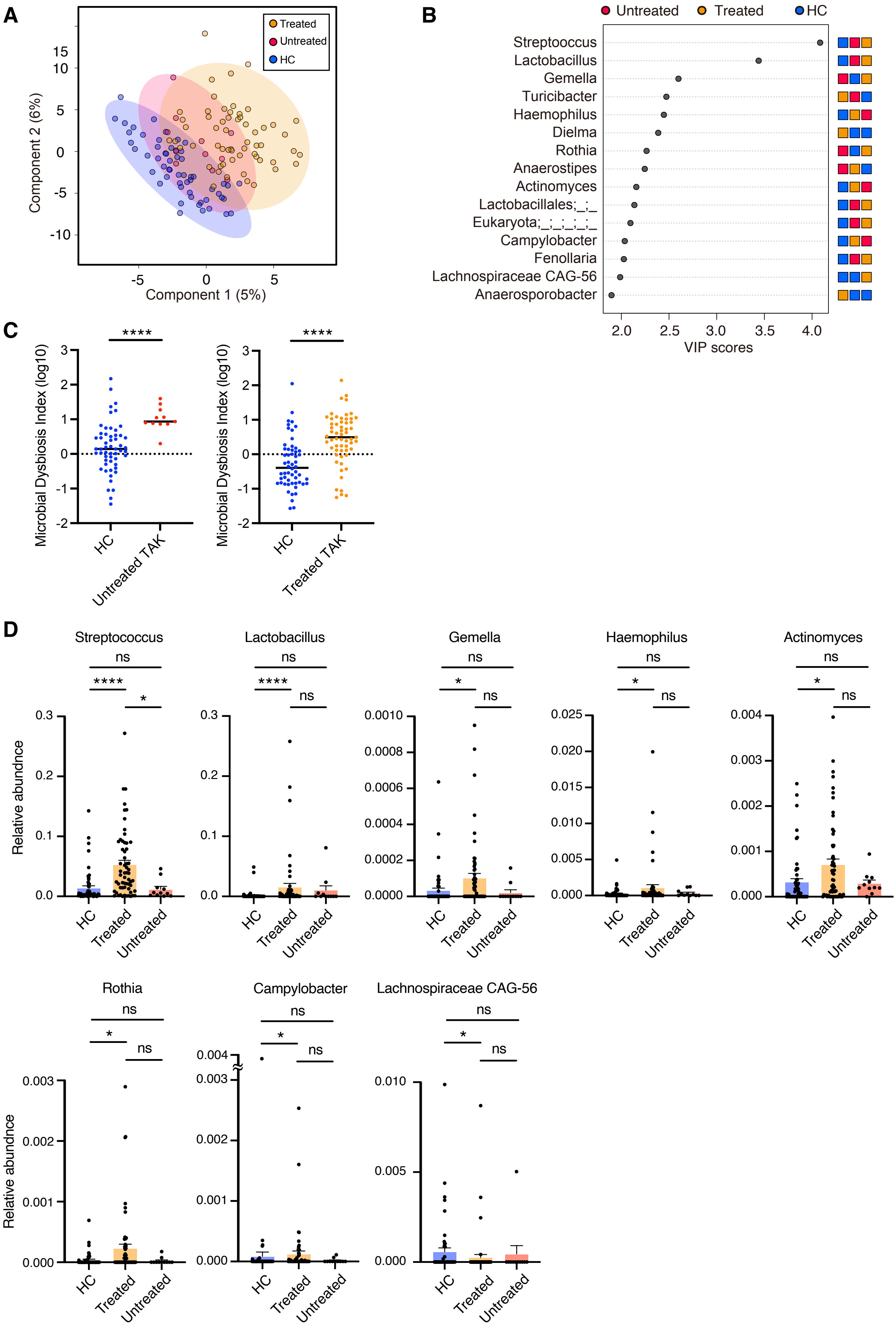

Supplementary Figure S5

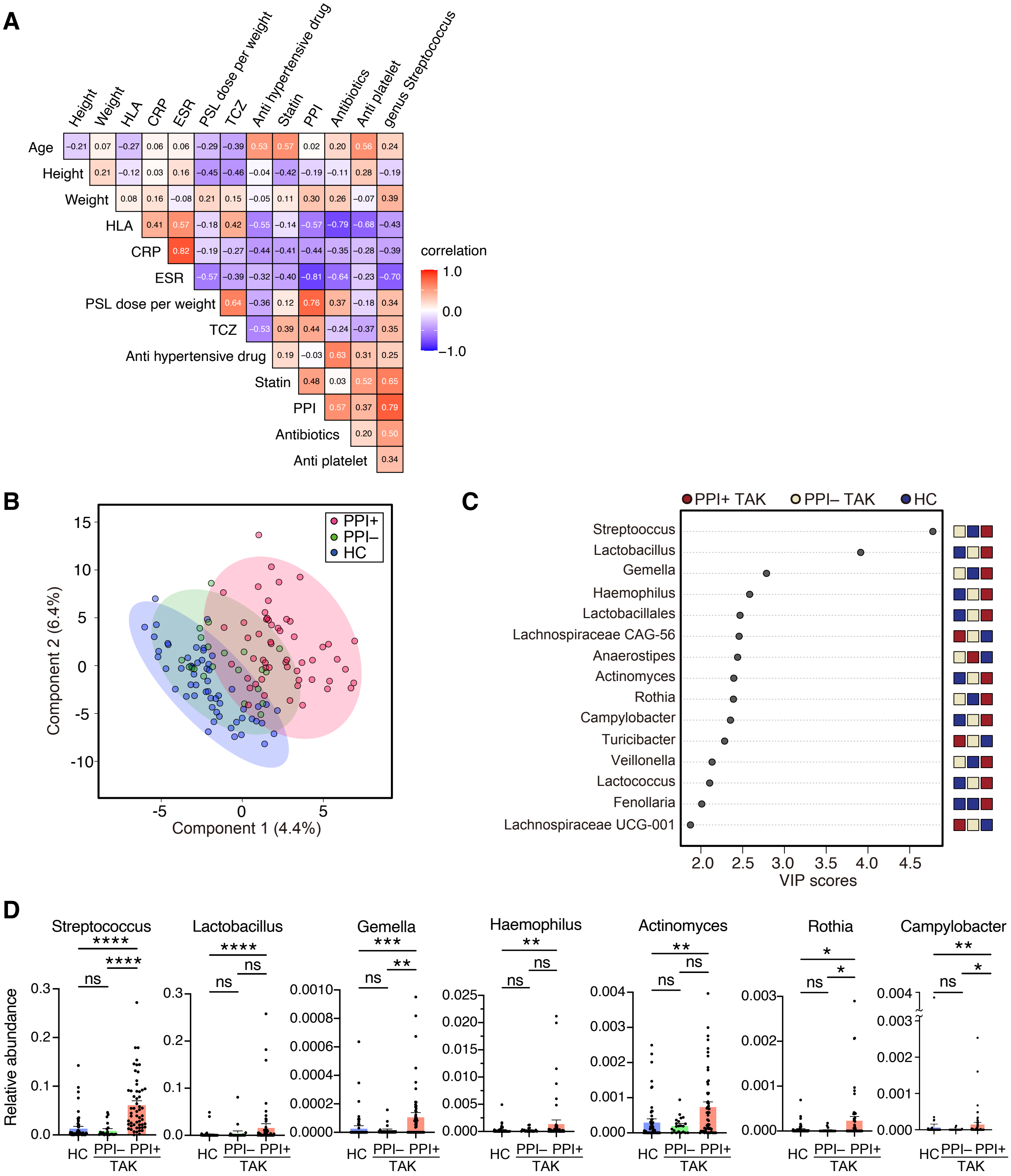

Supplementary Figure S6

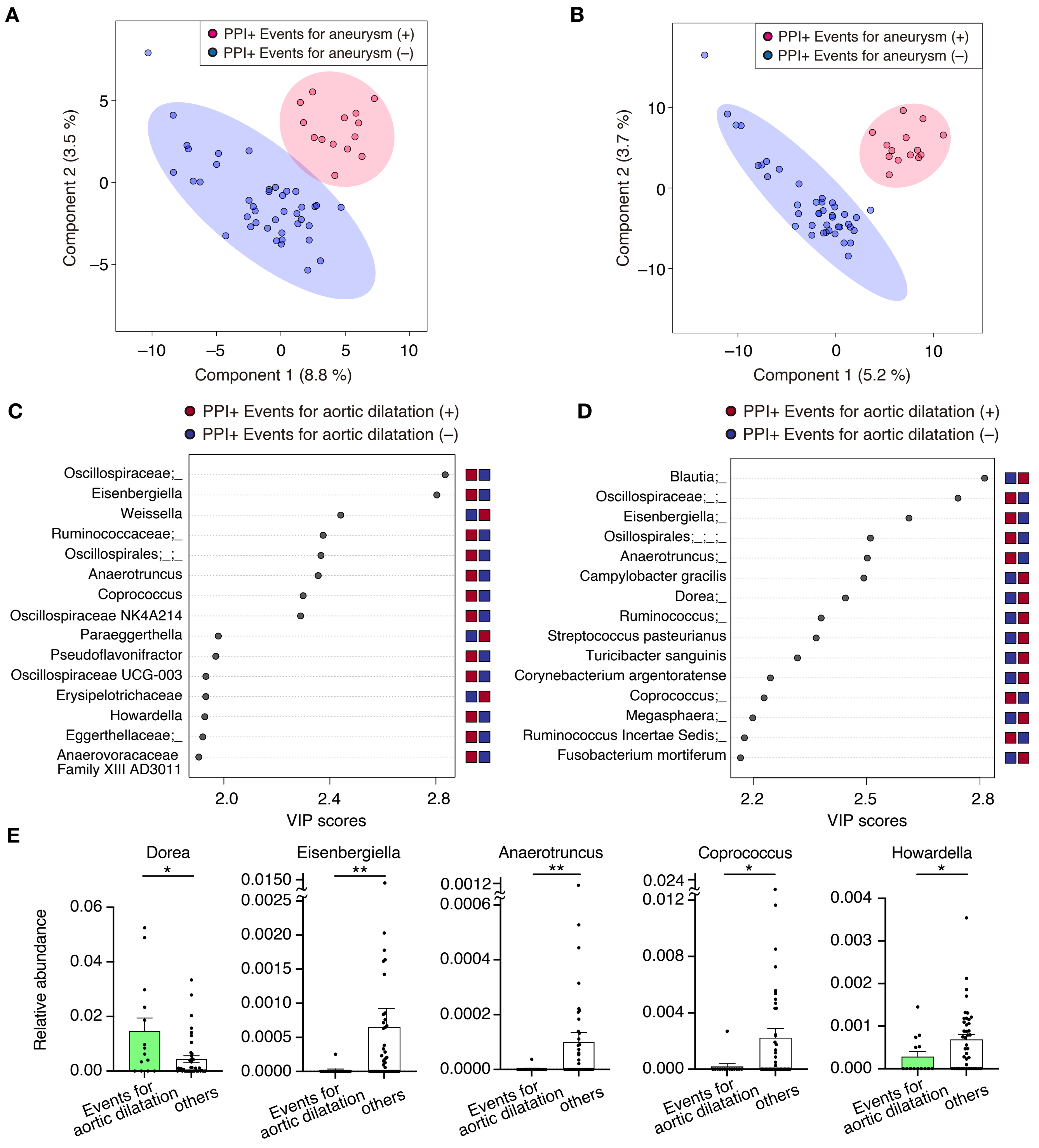

Supplementary Figure S7

A

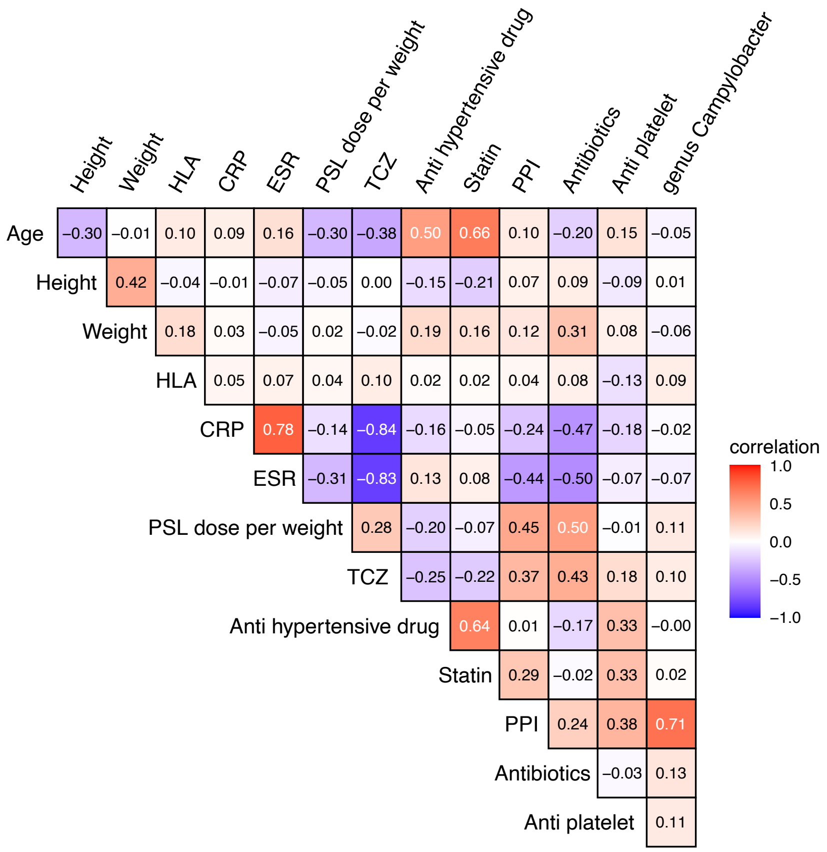

Supplementary Figure S8

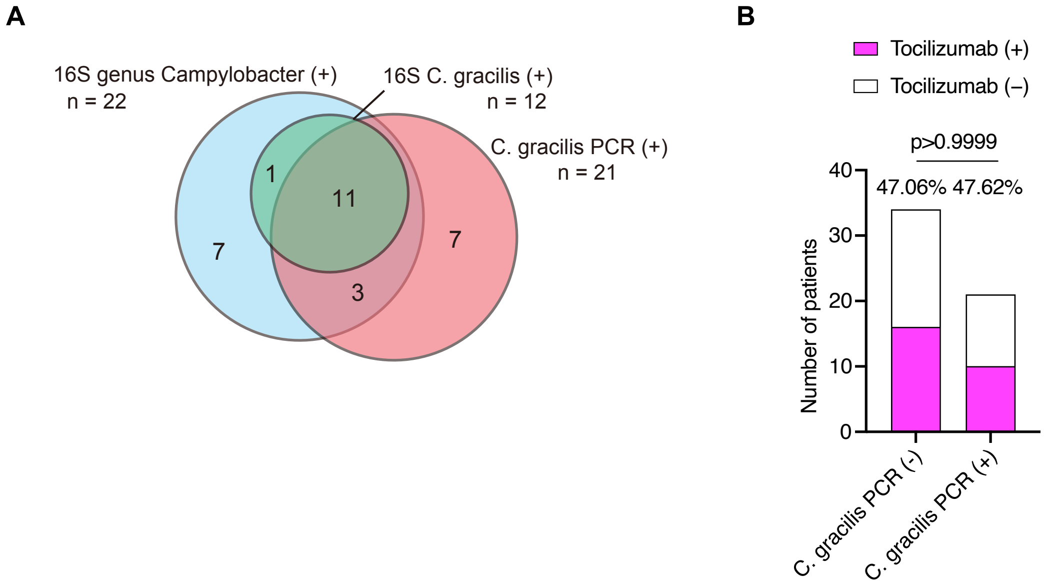

Supplementary figure legends

Supplementary Figure S1. Gut microbial composition in patients with TAK and HCs (related to Fig. 1). (**A**) and (**B**) Differences in gut microbial composition between TAK and HCs at the phylum level (**A**) and the family level (**B**). (**C**) and (**D**) Bar plots of relatively increased bacteria (**C**) and decreased bacteria (**D**) in patients with TAK compared with HCs. The bars show the mean ± standard error of the mean. Each dot represents an individual (n = 76 for patients with TAK and n = 56 for HCs). *$P < 0.05$, **$P < 0.01$.

Supplementary Figure S2. Gut microbial diversity and taxonomy in patients with active TAK (n = 14) and HCs (n = 56). (**A**) Alpha diversity (Shannon index, Faith's phylogenetic diversity, and numbers of operational taxonomic units [OTUs]) in patients with TAK and HCs. (**B**) partial least-squares discriminant analysis in patients with active TAK and HCs. (**C**) Variable importance of projection (VIP) score of component 1 in panel B. The red squares represent relatively increased bacteria and the blue squares represent relatively decreased bacteria. **(D)** and **(E)**, Differences in gut microbial composition between patients with active TAK and HCs at the phylum level (**D**) and the family level (**E**).

Supplementary Figure S3. Gut microbial composition in patients with active TAK and HCs. (**A**) Volcano plot of the relative abundance of the gut microbiota at the genus level in patients with active TAK and HCs. The red dots represent increased bacteria and the blue dots represent decreased bacteria in patients with active TAK compared with HCs. (**B**) and (**C**) Bar plots of relatively increased bacteria (**B**) and decreased bacteria (**C**) in patients with active TAK compared with HCs. (**D**) Microbial dysbiosis index in patients with TAK and HCs (analysed by the Mann–Whitney U test). In the bar plots, data are shown as the mean ± standard error of

the mean. Each dot represents an individual (n = 14 for patients with active TAK and n = 56 for HCs). **P* < 0.05; ***P* < 0.01; ****P* < 0.001; *****P* < 0.0001.

Supplementary Figure S4. Gut microbial composition in treated and untreated TAK patients and HCs. (**A**) partial least-squares discriminant analysis in treated and untreated TAK patients and HCs. (**B**) Variable importance of projection (VIP) score of component 1 in panel A. (**C**) Microbial dysbiosis index analyzed by the Mann–Whitney U test. (**D**) Bar plots of relative abundance of bacteria in treated and untreated TAK patients and HCs. In the bar plots, data are shown as the mean ± standard error of the mean. Each dot represents an individual (n = 56 for HCs, n = 59 for treated TAK patients, and n = 11 for untreated TAK patients). ns, not significant. **P* < 0.05; *****P* < 0.0001.

Supplementary Figure S5. Effect of proton pump inhibitors (PPIs) on gut microbiota taxonomy. (**A**) Correlation coefficient of clinical parameters and the relative abundance of the genus *Streptococcus* in patients with active TAK. (**B**) partial least-squares discriminant analysis among patients with TAK taking PPIs, patients with TAK without PPIs, and HCs (without PPIs). (**C**) Variable importance of projection (VIP) score of component 1 in panel B. The squares on the right side indicate the order of relative abundance in each group (left: lower abundance, right: higher abundance). (**D)** Bar plots of the relative abundance of each bacterium in patients with TAK taking PPIs, patients with TAK without PPIs, and HCs (without PPIs) (analysed by Dunn's multiple comparisons test). In the bar plots, data are shown as the mean ± standard error of the mean. Each dot represents an individual (n = 55 for patients with TAK and PPIs, n = 21 for patients with TAK without PPIs, and n = 56 for HCs). ns, not significant. **P* < 0.05; ***P* < 0.01; ****P* < 0.001; *****P* < 0.0001. HLA, human leukocyte antigen; CRP, C reactive protein; ESR, erythrocyte sedimentation rate; PSL, prednisolone; TCZ, tocilizumab.

Supplementary Figure S6. Gut microbiota taxonomy and the relationship between patients with TAK taking PPIs with or without aortic aneurysm-related events. (**A**) and (**B**) partial least-squares discriminant analysis in patients with TAK taking PPIs with or without aortic aneurysm-related events at the genus level (**A**) and the species level (**B**). (**C**) and (**D**) VIP score in component 1 of panel A (**C**) and panel B (**D**). The squares on the right side indicate the order of relative abundance in each group (left: lower abundance, right: higher abundance). (**E**) Bar plots of the relative abundance of bacteria in patients with TAK taking PPIs with or without aortic aneurysm-related events at the genus level. In the bar plots, data are shown as the mean ± standard error of the mean. Each dot represents an individual (n = 22 for patients with TAK with aortic aneurysm-related events and n = 33 for patients with TAK without these events). *$P < 0.05$, **$P < 0.01$.

Supplementary Figure S7. Effect of proton pump inhibitor (PPI) administration on the relative frequency of the genus *Campylobacter* in the gut microbiota of patients with TAK taking PPIs. HLA, human leukocyte antigen; CRP, C reactive protein; ESR, erythrocyte sedimentation rate; PSL, prednisolone; TCZ, tocilizumab; PPI, proton pump inhibitor.

Supplementary Figure S8. Clinical characteristics of patients with TAK taking PPIs who experienced aortic aneurysm-related events including prospective observations (related to Fig. 4). (**A**) Venn diagram showing the number of patients who were positive for the genus *Campylobacter* and *C. gracilis* by 16S rRNA sequencing and *C. gracilis* by specific PCR. (**B**) Relationship between the positivity of *C. gracilis* by specific PCR and the use of tocilizumab.
